# Author Correction: Single-cell profiling reveals heterogeneity and functional patterning of GPCR expression in the vascular system

**DOI:** 10.1038/s41467-019-09334-3

**Published:** 2019-03-28

**Authors:** H. Kaur, J. Carvalho, M. Looso, P. Singh, R. Chennupati, J. Preussner, S. Günther, J. Albarrán-Juárez, D. Tischner, S. Classen, S. Offermanns, N. Wettschureck

**Affiliations:** 1 Department of Pharmacology, Max Planck Institute for Heart and Lung Research, Ludwigstr 43, 61231 Bad Nauheim, Germany; 2 ECCPS Bioinformatics Facility, Max Planck Institute for Heart and Lung Research, Ludwigstr 43, 61231 Bad Nauheim, Germany; 3 ECCPS Deep sequencing platform, Max Planck Institute for Heart and Lung Research, Ludwigstr 43, 61231 Bad Nauheim, Germany; 4 Harvey Vascular Centre, Kerckhoff-Klinik, Benekestraβe 2–8, 61231 Bad Nauheim, Germany; 5 Medical Faculty, J.W. Goethe University, Theodor-Stern-Kai 7, 60590 Frankfurt, Germany

Correction to: *Nature Communications* 10.1038/ncomms15700; published online 16 June 2017

The original version of this Article omitted the following from the Acknowledgements:

‘This project was supported by CRC128/Project A03 of the Deutsche Forschungsgemeinschaft (DFG).’

This has not been corrected in either the PDF or HTML versions.

