# Supplementary material for: Single-cell profiling reveals heterogeneity and functional patterning of GPCR expression in the vascular system
Source: Nat Commun. 2017 Jun 16;8:15700. doi: 10.1038/ncomms15700 (PMC5481776; doi:10.1038/ncomms15700)
Supplement: Supplementary Information — Supplementary Figures and Supplementary Tables. [file ncomms15700-s1.pdf]

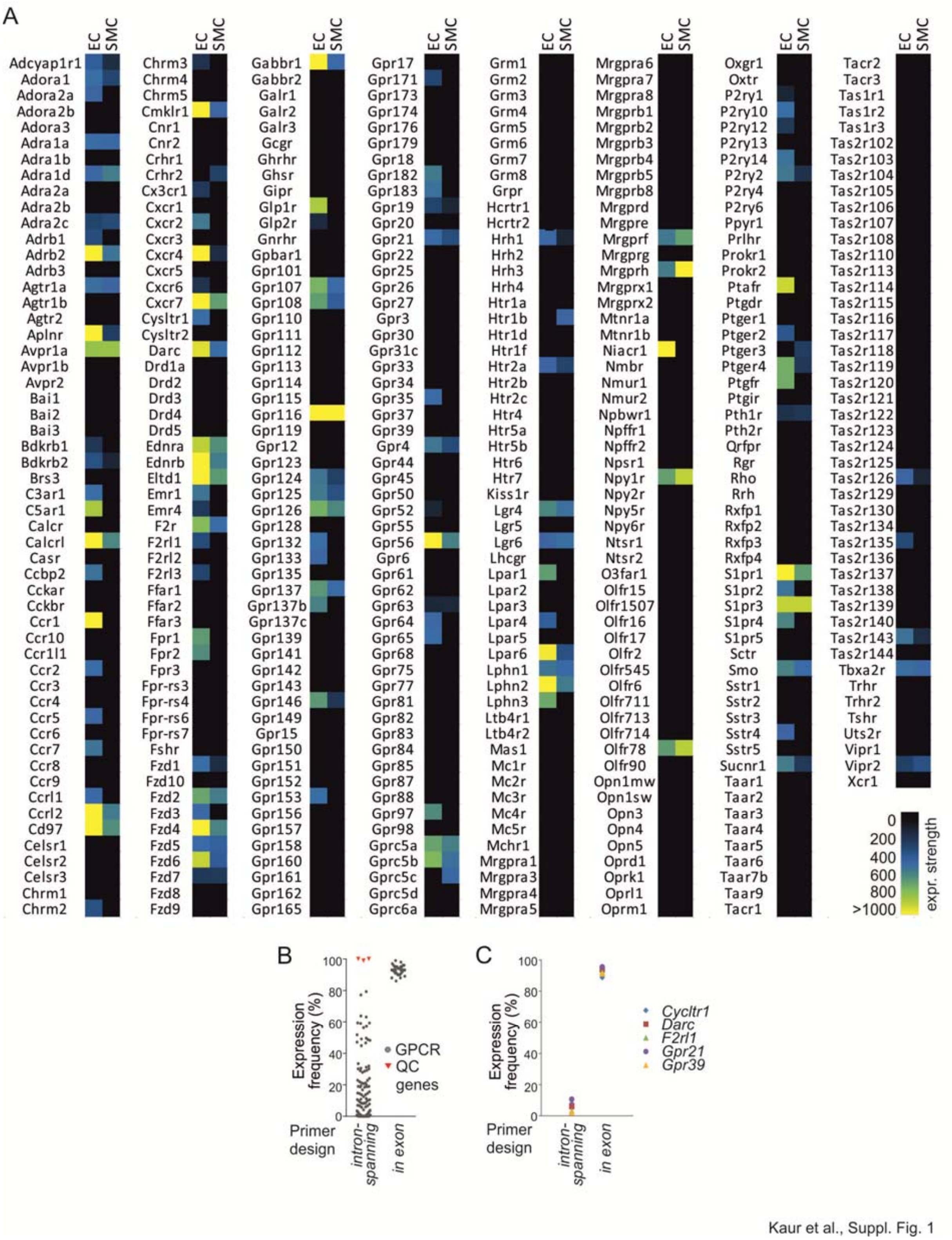

Supplementary Figure 1: A; GPCR expression profiles in 250-500 ng of pooled cell RNA obtained from freshly isolated murine EC (harvested from pulmonary or skeletal muscle vasculature) and SMC (harvested from aortae or skeletal muscle vasculature) as measured by the NanoString nCounter System. **B, C,** Expression frequency of selected GPCRs and three quality control (QC) genes (*Gapdh,*

Hprt, Actb) when analyzed using intron-spanning primer design or non-intron-spanning (in exon) primer design (each symbol one cell). Most intron-spanning primer pairs, with exception of the quality control genes *Actb*, *Hprt*, and *Gapdh*, showed amplification only in subpopulations of cells, whereas the 22 non intron-spanning primer pairs gave positive results in more than 90% of tested cells (B). This suggests that contamination with genomic DNA hampers the use of non-intron-spanning designs, and a direct comparison showed clearly lower expression frequencies for intron-spanning primers compared to their non-intron-spanning counterparts (C).

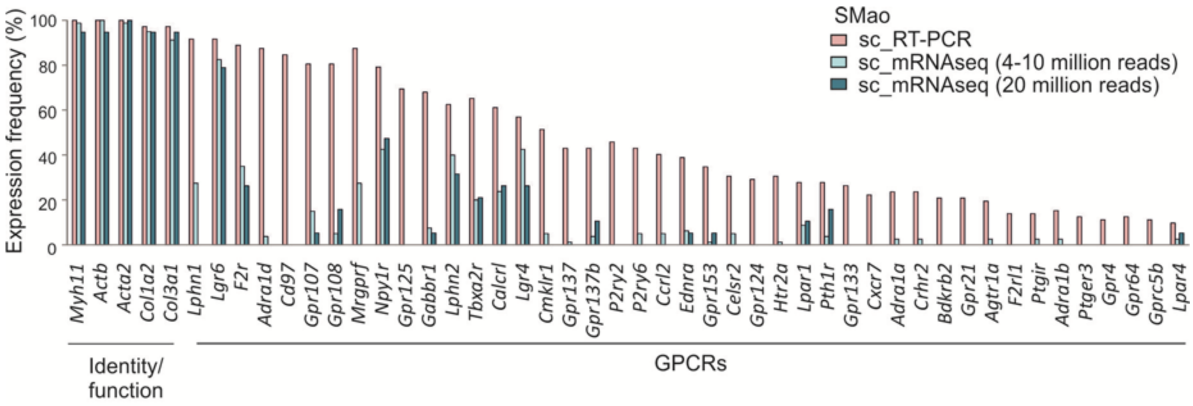

Kaur et al., Suppl. Fig. 2

Supplementary Figure 2: Comparison of GPCR expression frequency in freshly isolated vascular SMC of the aorta (SMao) as judged by single-cell RT-PCR (sc_RT-PCR) and two single-cell mRNA sequencing experiments (sc_mRNAseq) with different read depth. For full mRNA sequencing datasets, please see supplemental data set 1.

Suppl. Fig. 3: Alignment of sequencing results

```
Adra1a_F --------------------------------------------------ctgctggctgccattcttcctcgtgatgcccattgggtccttc---------
Amplicon gagaagaaagccgccaagacgctgggcattgtggtgggatgcttcgtcctctgctggctgccattcttcctcgtgatgcccattgggtccttcttcccgaat
Adra1a_R ------------gccaagacgctgggcattgtggtgggatgcttcgtcctc--------------------------------------------------

Adra1b_F ---------------------------------------------ctccaccctaaagcccccggacgccgtattcaaggtagtgttctggctgggc—
Amplicon atgttggctcccctcttcatcgctctcccacttggctccctgttctccaccctaaagcccccggacgccgtattcaaggtagtgttctggctgggcta
Adra1b_R atgttggctcccct-------------------------------------------------------------------------------------

Adra1d_F -----------------------------------------ggttctctgttccctcagctgaaaccatcag--------
Amplicon ttcgtcctgtgctggttccccttttcttcgtcctgcctctgggttctctgttccctcagctgaaaccatcagagggcgtc
Adra1d_R ttcgtcctgtgctggttccccttttct-----------------------------------------------------

Agtr1a_F -------------------------------------------------------gtgtctgagaccaactcaacccaga
Amplicon taactcacagcaaccctccaagaaagccatcaccagatcaagtgcattttgaacagtgtctgagaccaactcaacccaga
Agtra1a_Rtaactcacagcaaccctccaagaaagccatca------------------------------------------------

Bdkrb2_F ---------------------------------------------------tgcccaccgcggcctcctttggcatcgaaatgttcaacgtcaccac-----
Amplicon aaatgccctgctcctggaagctactcgggtttctgtcggtgcatgagcccatgcccaccgcggcctcctttggcatcgaaatgttcaacgtcaccacacaag
Bdkrb2_R --atgccctgctcctggaagctactcgggtttctgtcggtgcatgagcccat--------------------------------------------------

Calcrl_F -----------------------------------------------ctgggacggatggctatgctggaatgacgttg
Amplicon atgcaggaccccattcaacaagcagaaggcctttactgcaataggacctgggacggatggctatgctggaatgacgttg
Calcrl_R atgcaggaccccattcaacaagcaga-----------------------------------------------------

Ccrl2_F  -----------------------------------------------agcctccgatggataactacacagtggccc
Amplicon caagcaacctgcctcaaacgacgctgttttgtccggtgagcaaggacagcctccgatggataactacacagtggccc
Ccrl2_R  caagcaacctgcctcaaacgacgc-----------------------------------------------------

Cd97_F   ----------------------------------------ggacc-aaggctggaattgatcaccaaggtggg
Amplicon tgaccagctttgccatcctaatggctcagtaccatgtgcaggacccaaggctggaattgatcaccaaggtggg
Cd97_R   tgaccagctttgccatcctaatggctcagtacc----------------------------------------

Celsr2_F -----------------------------------------------tggtgacaactgtacaaatgtgtgtgacctgaaccca
Amplicon agcaatgactgggacagctattcttgtagctgtgttctaggttactatggtgacaactgtacaaatgtgtgtgacctgaaccca
Celsr2_R agcaatgactgggacagctattcttgtag-------------------------------------------------------
```

Suppl. Fig. 3 (cont.): Alignment of sequencing results

```
Cmklr1_F ---------------------------------------------------------------ctccaaagagatggagtacgacgcttacaacgac
Amplicon gtaacagaccagccaaggacca-ggactggagttctgttctacaacggtgaacagtgaaaggtctccaaagagatggagtacgacgcttacaacgac
Cmklr1_R gtaacagaccagccnaggaccaaggac-ggag-----------------------------------------------------------------

Crhr2_F  -----------------------------------------------tttcaggtccctacacctactgcaacacgaccttgga
Amplicon gtggacacttttggagcagtactgccacaggaccacaactgggaatttttcaggtccctacacctactgcaacacgaccttgga
Crhr2_R  gtggacacttttggagcagtactgccacaggac---------------------------------------------------

Cxcr7_F  -------------------------------------------------caaaccacagcccaggaagccctgaggtcacttggtcgctctcctcaagac
Amplicon acaaactgctcagcactgaaggagcctgcagcgctcaccgtcaggaaggcaaaccacagcccaggaagccctgaggtcacttggtcgctctcctcaagac
Cxcr7_R  acaaactgctcagcactgaaggagcctgcagcgctcaccgtcaggaag----------------------------------------------------

Ednra_F  ---------------------------------------------------gatgtgaaggactggtggctcttt
Amplicon cataggacctgcatgctcaacgccacgtccaagttcatggagttttaccaagatgtgaaggactggtggctcttt
Ednra_R  cataggacctgcatgctcaacgccacgtccaagt-----------------------------------------

F2r_F    ------------------------------------------------agaggacagatgctacggtga
Amplicon gcggtcccttgctgtcttcccgcgtccctatgagccagccagaatcagagaggacagatgctacggtga
F2r_R    gcggtcccttgctgtcttcccgc----------------------------------------------

F2rl1_F  ------------------------------------------------ggaccgagaaccttgcaccgggacgcaacaacagtaaaggaagaagtct-----------
Amplicon ggctgctgggaggtatcacccttctggcggcctcggtctcctgcagccggaccgagaaccttgcaccgggacgcaacaacagtaaaggaagaagtcttattggcagat
F2rl1_R  -------------------------------ctcggtctcctgcagccggaccgagaaccttgcaccgggacgcaacaaca---------------------------

Gabbr1_F --------------------------------------------------tcgtgggacttttctatgagaccgaagcccg
Amplicon agatccagctgtgcctgttaaaaacctgaagcgtcaagatgctcgaatcatcgtgggacttttctatgagaccgaagcccg
Gabbr1_R agagccagctgtgcctgntaaaaacctgaagcgtc----------------------------------------------

Gpr107_F -----------------------------------------------cgcagggctttccgattgaaggctgggctgttgta
Amplicon gtctctctcgttggtgttccatgcaatcgactaccactacatctcctcgcagggctttccgattgaaggctgggctgttgta
Gpr107_R gtctctctcgttggtgttcca-gcaa--------------------------------------------------------

Gpr108_F ------------------------------------------------tcactgtgatgatccgggagaagaatcca
Amplicon tccacaactgtcacaactccatcccaggccaggagcagccattcgacctcactgtgatgatccgggagaagaatcca
Gpr108_R nccacaactgtcacaac------------------------------------------------------------
```

Suppl. Fig. 3 (cont.): Alignment of sequencing results

```
Gpr124_F  ----------------------------------------------------------ttccttgtcgtcactgctctggat
Amplicon  tcacgctcaccaactaccaaatggtttgtcaagcggtgggcatcactctgcactactcttccttgtcgtcactgctctggat
Gpr124_R  tcacgctcaccaactaccaaatggtt-gtca---------------------------------------------------

Gpr125_F   ------------------------------------------------------------ttattctacccttgccacggt
Amplicon  tgggaggaataacccagaccagaaatgccagcgtctgtcaagcagttgggatcattcttcattattctacccttgccacggt
Gpr125_R  tgggaggaataacccagaccagaaatgccag---------------------------------------------------

Gpr133_F  --------------------------------------------------------------agaacggtgggt-----
Amplicon  tccgcttgcccaataaatccctctcagaggaaacggcgctgaacctcacagagaccttcttaagaacggtgggtgaggt
Gpr133_R  tccgcttgcccaataaatccctctcagag--------------------------------------------------

Gpr137_F  ----------------------------------------------------ctctggcagttggtatggtgccatcgga
Amplicon  gctcctgggagcatagccggagtgagagcaccagcatgtccggcagcctgggctctggcagttggtatggtgccatcgga
Gpr137_R  gctcctgggagcatagccggagtgagagc--------------------------------------------------

Gpr137b_F --------------------------------------cagtgtgtcaggtaactgccattggtgtcaccgtcatctt-
Amplicon  tgtccctggccaacatctacttggagtcaaagggctcatcagtgtgtcaggtaactgccattggtgtcaccgtcatcttg
Gpr137b_R-gtccctggccaacatctac-tggagtcaaa-------------------------------------------------

Gpr153_F  --------------------------------------------------ccccgacatggtattggagcgctctctt
Amplicon  gccaacgacgaagattctgacaatgagaccagtctagagggcagcatctcccccgacatggtattggagcgctctctt
Gpr153_R  -ccaacgacgaagattctgacaatgagaccagtc--------------------------------------------

Gpr21_F   ------------------------------------------------------atgcagaatcacagctgttcagaggagactcattacaactcctgc-
Amplicon  gcattgcaaggctttcggttaaggatgtattgtggcttttgtttggatttcagcatgcagaatcacagctgttcagaggagactcattacaactcctgct
Gpr21_R   -----gcaaggctttcggttaaggatgtattgtggcttttgtttggatttcagca---------------------------------------------

Gpr4_F    --------------------------------------------------tagcagtcgcagctctcaatgcagttgtgagtggaacaacttcatctccact
Amplicon  cgggaccaagtcagagacgccggtcgcccagcccagccgcggagcaggcctagcaatctcagctctcaatgcagtcgtgagtggaacaacttcatctccactcctcag
Gpr4_R    ----------------------------------agccgcggagcaggcctagcagtcgcagctctcaatgcagttgtgagtgaa---------------

Gpr64_F   ------------------------------------------------------tgtgtattctgggacttgggcagaa
Amplicon  gtgacagtcgcactgaaacacatcaacccaagtccggatgacttaactgtgaaatgtgtattctgggacttgggcagaa
Gpr64_R   gtgacagtcgcactgaaacacatcaacc---------------------------------------------------
```

Suppl. Fig. 3 (cont.): Alignment of sequencing results

```
Gprc5b_F -----------------------------------------------------ccgatcagcagtgggcttttccaatggaagcttggagcaaagatc-
Amplicon cgggcctacatggagaacaaggccttctcaatggatgaacataacgcagctctccgatcagcagtgggcttttccaatggaagcttggagcaaagatct
Gprc5b_R cgggcctacatggagaacaaggccttctcaatggatgaacataacgca---------------------------------------------------

Htr2a_F  --------------------------------------------------ggtaccggtggcctttgcccagcaagctctgt
Amplicon gctgctgggtttccttgtcatgcccgtgtccatgttaaccatcctgtatgggtaccggtggcctttgcccagcaagctctgt
Htr2a_R  gctgctgggtttccttgtcatgcccgtgtccatgt-----------------------------------------------

Lgr4_F   ------------------------------------------------gtaattctatttctgttatcccggatggagca-
Amplicon ctcaggctattaaagcccttcccagccttaaagagctgggatttcacagtaattctatttctgttatcccggatggagcat
Lgr4_R   ctcaggctattaaagcccttcccagccttaa--------------------------------------------------

Lgr6_F   ---------------------------------------------------gctgcatctacataacaaccgcatccagcatg
Amplicon tatccgccacatccctgactatgccttccagaacctcaccagtcttgtggtgctgcatctacataacaaccgcatccagcatg
Lgr6_R   tatccgccacatccctgactatgccttccagaacc------------------------------------------------

Lpar1_F  -----------------------------------------------gactgtggtcattgtgcttggtgcctttattgtctgctggactccgg
Amplicon agttctggacccaggaggaatcgggacaccatgatgagccttctgaagactgtggtcattgtgcttggtgcctttattgtctgctggactccgg
Lpar1_R  agttctggacccaggaggaatcgggacaccatgatgagccttc---------------------------------------------------

Lpar4_F  --------------------------------------------------------ttctctcatctagcacactctttcttgggcactcaattgaggaac
Amplicon agtgcgagttgccagtttacacgtttattagctaactatctacaggcatgagcacattctctcatctagcacactctttcttgggcactcaattgaggaactctctga
Lpar4_R  --------------------acgtttattagctaactatctacaggcatgagcacattctctcatctag--------------------------------

Lphn1_F  -----------------------------------------ggtgaaagttgtcttcattctctacaacaacctgggcctcat
Amplicon tctccgccaacacc-atcaagcagaacagccgcaacggtgtggtgaaagttgtcttcattctctacaacaacctgggcctctt
Lphn1_R  tctccgccaacaccgatcaagcagaacagccgcaa------------------------------------------------

Lphn2_F  -------------------------------------------gaca-ccttctgagagctgaggctttggaatcctgga
Amplicon gaagacatgcagggcttaccttaaggcaattgtggacacggtagacaaccttctgagagctgaggctttggaatcctgga
Lphn2_R  gaagacatgcagggcttaccttaaggcaat--------------------------------------------------

Mrgprf_F -----------------------------------------------------tgtcctggtatgagcgaggctcggga
Amplicon agatggccggaaactgttcatgggaagctcactccaccaaccagaacaagatgtgtcctggtatgagcgaggctcggga
Mrgprf_R aga-ggccggaaactgttcatgggaagctcacgcc--------------------------------------------
```

Suppl. Fig. 3 (cont.): Alignment of sequencing results

```
Npy1r_F   ---------------------------------------------------------aggagaaacaacatgatggacaagatccggg
Amplicon  ttcggcccactctgctttatattcatatgctacttcaagatatacattcgcttgaaaaggagaaacaacatgatggacaagatccggg
Npy1r_R   ttcggcccactctgctttatattcatatgctacttcaa---------------------------------------------------

P2ry2_F   --------------------------------------------------------gacctggaaccctggaatagcacc
Amplicon  gagcatcctcaccacctcaagagcaggagctgatcaggtccagggcaatggcagcagacctggaaccctggaatagcacc
P2ry2_R   gagcatcctcaccacctcaagagcaggagctgatcag--------------------------------------------

P2ry6_F   -------------------------------------------------------cgagcataggaaaggctgacaggcag
Amplicon  ccaaatctggcacttcctcctaaaacatcttccatcttgcatgagacagactctccgagcataggaaaggctgacaggcag
P2ry6_R   ccaaatctggcacttcctcctaaaacatcttcc------------------------------------------------

Ptger3_F  ---------------------------------------------ctattgataatgatgttgaaaatgatcttcaatcagatgtcggttgagc
Amplicon  cagctcatggggatcatgtgtgtgctgtccgtctgttggtcgccgctattgataatgatgttgaaaatgatcttcaatcagatgtcggttgagc
Ptger3_R  cagctcatggggatcatgtgtgtgctgtccgtctgttggtcgccgct-----------------------------------------------

Ptgir_F   --------------------------------------------------------ccgaggcttcactcaggccatcgcc
Amplicon  attctgctggccctcatgaccgtcatcatggccgtgtgctccctgcctctcatgatccgaggcttcactcaggccatcgcc
Ptgir_R   attctgctggccctcatgaccgtcatcatggccgtgtgc-------------------------------------------

Pth1r_F   -------------------------------------------------ctcaacttcatcctctttatcaacatcatccgggtgct
Amplicon  tgggcacaagaagtggatcatccaggtgcccatcctggcatctgttgtgctcaacttcatcctctttatcaacatcatccgggtgct
Pth1r_R   tgggcacaagaagtggatcatccaggtgcngatcc----------------------------------------------------

Tbxa2r_F  --------------------------------------------------------ttcatcatgcagactttgttgcagacaccacct-
Amplicon  ttcagctcgtgggcatcatggtggtggccacggtgtgttggatgcccttgctggtcttcatcatgcagactttgttgcagacaccacctg
Tbx2ar_R  ttcagctcgtgggcatcatggtggtggccacggtgtgttggatgc---------------------------------------------
```

Supplementary Figure 3: Alignment of sequences obtained by sequencing of single-cell amplicons with forward (F) and reverse (R) primers. Non-matching base pairs are highlighted in yellow. The BLAST results for the obtained sequences are shown in Supplemental data set 2.

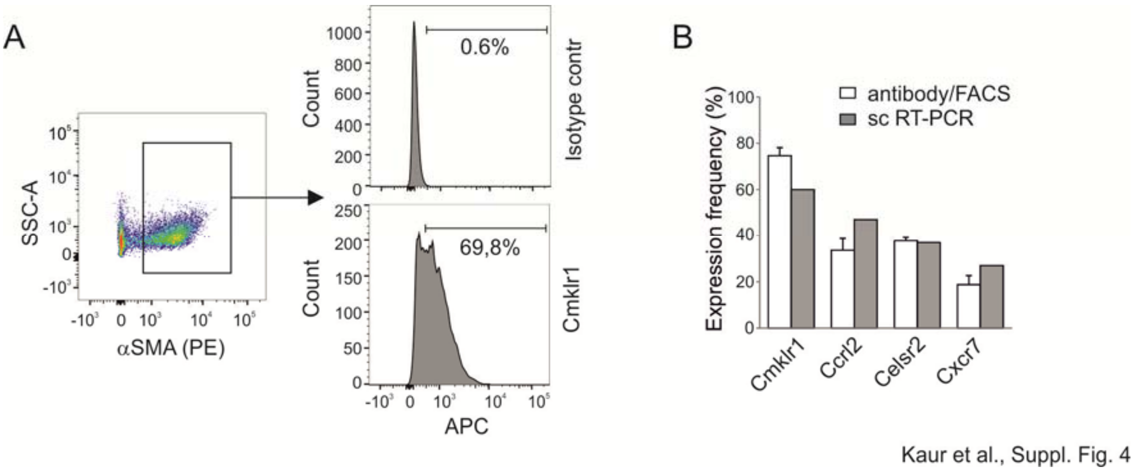

Supplementary Figure 4: A, B, Flow cytometric analysis of GPCR expression: Example of the gating strategy (A) and statistical evaluation (B) of GPCR expression frequency in individual SMao as judged by flow cytometry (antibody/FACS) or single-cell RT-PCR (sc RT-PCR)(n=3-4). Data in B are means ± SEM.

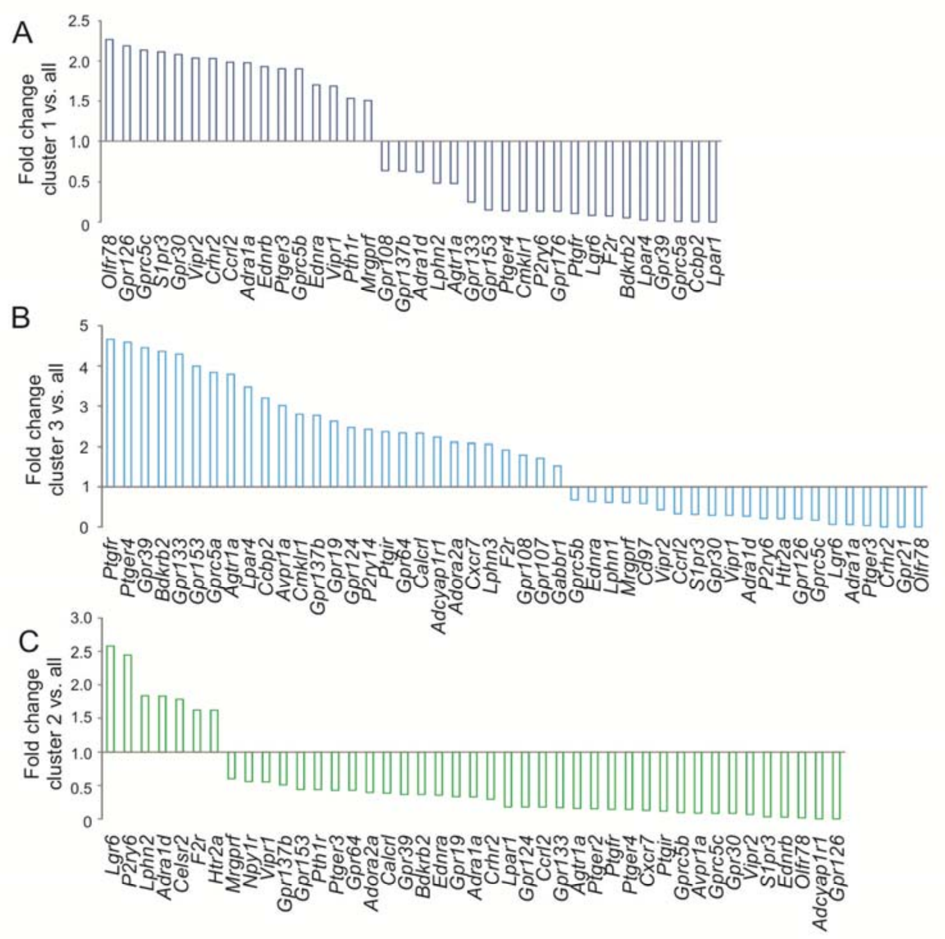

D

% of all	*Adcyap1r1*	*Adora2a*	*Adra1a*	*Adra1b*	*Adra1d*	*Agtr1a*	*Avpr1a*	*Bdkrb2*	*Calcrl*	*Ccbp2*	*Ccrl2*	*Cd97*	*Celsr2*
SMao	3	7	25	15	95	22	0	23	67	5	47	90	37
SMsk	82	39	89	5	89	35	61	16	96	0	86	96	21
SMmes	28	24	62	28	79	45	41	0	69	0	62	90	7
SMub	64	4	8	12	8	44	36	16	64	24	44	40	8

% of all	*Cmklr1*	*Crhr2*	*Cxcr7*	*Darc*	*Ednra*	*Ednrb*	*Emr1*	*F2r*	*F2rl1*	*Gabbr1*	*Gpr107*	*Gpr108*	*Gpr124*
SMao	60	25	27	0	43	2	2	97	17	77	90	90	35
SMsk	16	89	93	5	98	42	0	4	2	75	86	79	91
SMmes	21	45	52	0	76	41	0	34	0	66	72	62	86
SMub	56	0	88	16	32	16	12	72	0	60	76	80	84

% of all	*Gpr125*	*Gpr126*	*Gpr133*	*Gpr137*	*Gpr137b*	*Gpr153*	*Gpr176*	*Gpr19*	*Gpr21*	*Gpr30*	*Gpr39*	*Gpr4*	*Gpr64*
SMao	78	0	30	52	52	40	10	8	23	0	10	13	13
SMsk	65	93	7	68	58	30	2	23	44	49	0	2	28
SMmes	52	21	45	62	48	10	7	14	17	10	3	3	10
SMub	36	0	68	36	68	76	8	12	0	12	16	0	0

% of all	*Gpr83*	*Gprc5a*	*Gprc5b*	*Gprc5c*	*Hrh2*	*Htr2a*	*Lgr4*	*Lgr5*	*Lgr6*	*Lpar1*	*Lpar2*	*Lpar4*	*Lphn1*
SMao	2	8	13	10	3	35	67	0	98	33	2	12	100
SMsk	0	0	93	100	4	40	63	0	32	2	0	0	100
SMmes	0	3	69	69	7	14	48	0	17	10	0	3	86
SMub	8	28	60	12	0	4	12	12	0	84	8	12	64

% of all	*Lphn2*	*Lphn3*	*Mrgprf*	*Npy1r*	*Olfr78*	*P2ry14*	*P2ry2*	*P2ry6*	*Prokr1*	*Ptger1*	*Ptger2*	*Ptger3*	*Ptger4*
SMao	72	2	90	85	2	5	52	48	2	10	3	15	3
SMsk	32	2	96	100	100	2	54	7	0	2	16	32	2
SMmes	38	21	79	83	66	10	14	7	0	0	0	10	24
SMub	12	12	68	68	0	16	20	0	16	16	12	4	44

% of all	*Ptgfr*	*Ptgir*	*Pth1r*	*S1pr1*	*S1pr3*	*Tbxa2r*	*Tpra1*	*Vipr1*	*Vipr2*
SMao	3	17	33	5	8	72	60	5	7
SMsk	4	40	81	4	100	60	72	23	65
SMmes	0	76	24	7	86	48	28	0	31
SMub	28	56	24	0	8	28	48	12	4

Kaur et al., Suppl. Figure 5

Supplementary Figure 5: Differential GPCR expression in different SMC types. A-C, Fold difference in gene expression in cluster 1 cells (mainly SMsk and SMmes) (A), cluster 3 cells (mainly SMub) (B), or cluster 2 cells (mainly SMao) (C) compared the rest of all SMC. **D,** GPCR frequencies for all SMC types are depicted as horizontal bars (only GPCRs with expression frequency >3% in at least one cell type are shown).

% of all	Adcyap1r1	Adora1	Adora2a	Adra1a	Adra1b	Adra1d	Agtr1a	Avpr1a	Bdkrb2	C3ar1	C5ar1	Calcrl	Ccbp2	Ccr10	Ccr1	Ccr2	Ccr3	Ccr4	Ccr5	Ccr6	Ccr7
SMao	3	0	7	25	15	95	22	0	23	0	2	67	5	0	0	0	0	0	0	0	0
SMaoAged	13	0	3	16	22	78	9	0	9	0	0	50	0	0	0	0	0	0	0	0	0
SMaoApo0w	6	0	3	6	15	91	15	9	6	0	0	47	0	0	0	0	0	0	0	0	0
SMaoApo16w	10	0	14	24	20	90	36	14	16	0	0	88	0	0	0	0	0	0	0	0	0
SMsk	82	2	39	89	5	89	35	61	16	0	0	96	0	0	0	2	0	0	0	0	0
SMmes	28	0	24	62	28	79	45	41	0	0	0	69	0	0	0	0	0	0	0	3	0
SMub	64	0	4	8	12	8	44	36	16	0	0	64	24	0	0	0	0	0	4	0	0
ECsk	3	0	53	0	3	0	0	0	13	0	0	98	20	0	0	0	0	0	0	0	8
EClu	0	0	25	0	0	0	0	0	10	0	0	100	73	0	0	0	0	0	0	0	4
ECluLPS	0	0	12	0	0	0	0	0	12	0	0	92	88	0	0	0	0	0	0	0	8
ECbr	0	0	2	0	0	0	0	0	0	0	0	87	0	0	0	0	0	0	0	0	0
ECbrLPS	0	0	23	0	0	0	0	0	9	0	9	64	0	0	9	0	0	0	0	0	0
ECao	0	0	18	0	0	36	0	0	9	0	9	82	0	0	18	0	0	0	0	0	0
ECaoApo16w	0	0	19	6	0	0	6	0	0	0	0	38	0	0	0	0	0	0	6	0	6
CD4 T cell	0	0	39	0	0	0	0	0	0	0	1	39	0	1	5	37	1	44	24	6	50
Macrophage	0	3	8	8	0	0	0	0	0	59	71	49	0	0	55	45	6	0	71	0	1
Neutrophil	4	0	0	0	0	4	4	0	4	13	74	43	0	0	43	0	0	0	4	0	0

% of all	Ccr8	Ccr9	Ccrl1	Ccrl2	Cd97	Celsr2	Chrm2	Chrm3	Cmklr1	Cnr2	Crhr2	Cx3cr1	Cxcr1	Cxcr2	Cxcr3	Cxcr4	Cxcr5	Cxcr6	Cxcr7	Cysltr1	Darc
SMao	0	0	0	47	90	37	0	0	60	0	25	0	0	0	2	2	0	0	27	2	0
SMaoAged	0	0	0	41	88	47	3	0	44	0	28	0	0	0	0	0	0	3	34	0	0
SMaoApo0w	0	0	0	32	88	26	0	3	59	0	18	0	0	0	0	0	0	0	38	0	0
SMaoApo16w	0	0	0	74	90	40	8	6	90	0	44	0	0	0	0	8	0	0	78	4	0
SMsk	0	0	2	86	96	21	0	0	16	0	89	0	0	0	0	2	0	0	93	0	5
SMmes	0	0	0	62	90	7	0	0	21	0	45	0	0	0	0	3	0	0	52	3	0
SMub	0	0	0	44	40	8	0	0	56	4	0	4	0	0	0	4	0	0	88	0	16
ECsk	0	0	0	40	98	18	3	3	18	0	0	0	0	0	0	48	0	0	78	15	18
EClu	0	0	0	92	98	56	6	21	13	0	0	0	0	0	0	56	0	0	69	0	2
ECluLPS	0	0	0	92	60	28	4	12	12	0	0	0	0	4	0	4	0	0	92	0	0
ECbr	0	0	0	60	98	6	0	0	10	0	0	0	0	0	0	2	0	0	69	2	0
ECbrLPS	0	0	0	64	77	5	0	0	0	0	0	0	0	0	0	0	0	0	86	0	68
ECao	0	0	0	27	100	0	0	9	45	0	18	0	0	0	0	45	0	0	82	0	0
ECaoApo16w	0	0	0	38	81	0	0	0	31	0	0	0	0	0	0	25	6	0	25	0	13
CD4 T cell	33	2	1	14	87	6	0	0	5	12	0	6	7	1	34	50	8	33	0	12	0
Macrophage	0	0	0	70	72	3	0	0	62	41	0	48	1	5	7	53	0	0	3	49	0
Neutrophil	0	0	0	35	70	0	0	4	4	26	0	13	0	26	0	57	0	0	4	0	0

% of all	Ednra	Ednrb	Eltd1	Emr1	Emr4	F2r	F2rl1	F2rl2	F2rl3	Fpr1	Fpr2	Gabbr1	Glp1r	Glp2r	Gpr107	Gpr108	Gpr111	Gpr114	Gpr116	Gpr124	Gpr125
SMao	43	2	0	2	0	97	17	0	0	0	0	77	0	0	90	90	0	0	2	35	78
SMaoAged	47	3	3	3	0	81	3	0	0	0	0	59	3	0	59	63	0	0	0	44	69
SMaoApo0w	50	21	0	0	0	79	6	0	0	0	0	44	0	0	65	74	3	0	6	56	68
SMaoApo16w	94	12	2	4	0	90	8	0	0	0	0	78	0	2	84	90	4	0	0	72	82
SMsk	98	42	0	0	0	4	2	0	4	0	0	75	0	0	86	79	2	2	2	91	65
SMmes	76	41	3	0	0	34	0	0	0	0	0	66	10	3	72	62	3	0	10	86	52
SMub	32	16	0	12	4	72	0	0	0	0	0	60	0	0	76	80	0	0	0	84	36
ECsk	5	75	98	0	0	48	10	0	3	3	0	90	3	0	80	60	0	0	100	30	33
EClu	2	19	96	2	0	31	0	0	0	2	0	92	98	0	71	65	0	2	98	69	81
ECluLPS	0	8	60	0	0	72	12	4	0	4	4	60	12	0	52	36	28	0	92	8	24
ECbr	2	0	98	0	0	13	0	0	0	0	0	65	0	0	44	56	2	0	100	90	46
ECbrLPS	0	5	100	0	0	18	0	0	0	9	9	45	0	0	68	50	23	0	100	59	18
ECao	9	45	100	18	0	73	9	36	0	0	0	55	0	0	91	45	0	0	91	18	27
ECaoApo16w	6	25	69	6	6	44	0	0	0	0	13	6	0	0	6	19	0	0	56	6	6
CD4 T cell	0	0	0	1	0	37	22	11	2	0	0	61	1	0	52	56	0	6	0	0	3
Macrophage	0	14	0	91	32	3	0	7	0	17	29	7	0	0	63	76	0	3	0	1	6
Neutrophil	4	0	9	9	0	0	0	0	0	30	74	17	0	0	17	48	0	0	4	0	4

% of all	Gpr126	Gpr132	Gpr133	Gpr137	Gpr137b	Gpr153	Gpr176	Gpr18	Gpr183	Gpr19	Gpr21	Gpr30	Gpr34	Gpr39	Gpr4	Gpr56	Gpr63	Gpr64	Gpr65	Gpr83	Gpr97
SMao	0	0	30	52	52	40	10	0	3	8	23	0	0	10	13	0	0	13	0	2	0
SMaoAged	3	0	16	38	47	25	0	0	0	34	19	3	3	3	3	6	3	3	0	3	0
SMaoApo0w	0	0	21	38	24	38	3	0	3	18	21	3	0	0	0	9	3	3	0	0	0
SMaoApo16w	14	0	42	70	72	66	20	0	2	28	30	4	0	10	12	14	0	26	0	0	0
SMsk	93	2	7	68	58	30	2	0	0	23	44	49	0	0	2	4	0	28	0	0	0
SMmes	21	3	45	62	48	10	7	0	0	14	17	10	0	3	3	3	0	10	0	0	0
SMub	0	0	68	36	68	76	8	0	4	12	0	12	0	16	0	0	0	0	0	8	0
ECsk	18	3	5	35	75	3	0	0	5	5	0	3	0	0	65	98	0	3	0	0	10
EClu	19	2	0	23	75	0	2	0	0	10	6	0	2	0	38	25	0	0	0	6	25
ECluLPS	4	4	4	8	40	0	0	0	0	4	0	0	0	0	8	44	4	0	0	0	28
ECbr	8	0	0	25	62	0	0	0	0	2	0	17	2	0	81	98	0	0	0	0	4
ECbrLPS	0	5	5	18	27	14	0	0	0	0	0	0	0	0	91	100	0	0	0	0	27
ECao	0	0	0	18	36	0	0	0	0	0	27	18	0	0	27	100	0	0	0	0	9
ECaoApo16w	6	0	6	13	31	38	0	0	0	0	0	0	0	0	0	56	0	0	19	0	0
CD4 T cell	0	80	2	22	7	0	0	43	54	10	0	0	10	0	0	4	0	0	31	4	4
Macrophage	25	51	1	51	64	1	4	21	45	6	0	0	13	0	0	10	0	0	59	0	0
Neutrophil	0	4	4	4	4	4	0	0	0	9	0	0	0	0	0	0	0	0	0	0	43

% of all	Gprc5a	Gprc5b	Gprc5c	Hrh2	Htr2a	Lgr4	Lgr5	Lgr6	Lpar1	Lpar2	Lpar3	Lpar4	Lphn1	Lphn2	Lphn3	Mrgprf	Npy1r	Olfr78	P2ry1	P2ry10	P2ry12
SMao	8	13	10	3	35	67	0	98	33	2	0	12	100	72	2	90	85	2	0	0	0
SMaoAged	16	22	25	3	28	63	6	88	25	0	0	6	84	63	0	69	91	13	3	0	0
SMaoApo0w	9	38	38	0	12	56	0	74	21	0	0	6	97	50	0	53	88	24	0	0	3
SMaoApo16w	10	72	82	8	34	64	4	92	34	2	6	10	96	86	4	78	78	36	0	0	0
SMsk	0	93	100	4	40	63	0	32	2	0	2	0	100	32	2	96	100	100	0	0	0
SMmes	3	69	69	7	14	48	0	17	10	0	0	3	86	38	21	79	83	66	0	0	0
SMub	28	60	12	0	4	12	12	0	84	8	4	12	64	12	12	68	68	0	0	0	0
ECsk	30	83	8	3	40	23	0	0	0	0	0	23	45	45	0	0	3	0	0	3	0
EClu	8	13	10	0	13	6	0	0	0	0	8	2	88	42	94	0	0	0	0	0	4
ECluLPS	24	64	16	0	12	8	0	0	4	0	8	0	20	64	12	0	0	0	0	8	0
ECbr	0	56	0	0	19	15	0	0	2	0	0	35	56	21	23	0	0	2	6	0	2
ECbrLPS	23	82	0	0	50	9	0	0	0	0	0	18	9	77	14	0	0	0	0	0	0
ECao	36	27	18	18	36	18	0	36	0	0	0	27	73	36	0	18	27	0	9	0	73
ECaoApo16w	0	19	0	6	13	6	0	6	0	0	0	13	13	13	0	0	19	19	0	6	0
CD4 T cell	4	0	0	13	1	3	0	0	0	6	0	0	49	0	0	0	0	0	1	60	0
Macrophage	2	12	13	2	12	7	0	0	8	2	0	0	5	6	0	0	0	0	7	1	18
Neutrophil	0	4	0	13	0	9	0	0	9	0	0	0	4	0	0	0	0	0	4	0	4

% of all	P2ry13	P2ry14	P2ry2	P2ry6	Prokr1	Ptafr	Ptger1	Ptger2	Ptger3	Ptger4	Ptgfr	Ptgir	Pth1r	S1pr1	S1pr3	Sucnr1	Tbxa2r	Vipr1	Vipr2	Xcr1	
SMao	0	5	52	48	2	0	10	3	15	3	3	17	33	5	8	0	72	5	7	0	
SMaoAged	0	13	50	25	0	0	3	0	25	0	3	22	53	13	22	0	69	9	3	0	
SMaoApo0w	0	6	26	24	0	0	9	9	24	3	0	15	21	6	26	0	53	3	12	0	
SMaoApo16w	0	14	56	72	2	2	30	8	36	16	6	58	56	22	62	0	64	6	10	0	
SMsk	0	2	54	7	0	0	2	16	32	2	4	40	81	4	100	2	60	23	65	0	
SMmes	0	10	14	7	0	0	0	0	10	24	0	76	24	7	86	0	48	0	31	0	
SMub	0	16	20	0	16	0	16	12	4	44	28	56	24	0	8	4	28	12	4	0	
ECsk	0	0	50	13	0	10	5	3	0	43	0	3	0	100	0	0	13	5	3	0	
EClu	4	0	8	2	0	0	2	0	0	0	2	0	0	100	0	0	19	0	0	0	
ECluLPS	0	0	68	8	0	12	0	8	0	0	0	0	0	100	4	0	0	4	4	0	
ECbr	0	0	33	2	0	0	2	0	0	0	2	0	6	98	0	0	27	0	0	0	
ECbrLPS	5	0	86	27	0	5	0	0	0	0	0	0	0	100	5	0	0	0	0	0	
ECao	0	0	55	9	27	0	0	0	0	27	0	0	18	100	0	0	45	0	0	0	
ECaoApo16w	0	0	6	0	0	6	0	0	0	6	0	0	6	88	19	0	13	0	0	6	
CD4 T cell	0	2	0	1	0	1	0	13	1	54	0	15	0	80	1	0	27	20	1	0	
Macrophage	25	14	28	74	14	68	0	13	1	54	0	25	0	26	0	0	0	1	0	0	
Neutrophil	4	4	4	9	4	30	0	0	4	9	0	17	0	9	4	0	0	0	0	0	

Kaur et al., Suppl. Figure 6

Supplementary Figure 6: GPCR frequencies for all investigated subtypes of SMC and EC as well as select immune cell types are depicted as horizontal bars. SMao/SMsk/SMmes/SMub, SMC from aorta (ao), skeletal muscle vasculature (sk), mesenteric vessels (mes), or urinary bladder (ub) (age: 2-4 months); Aged, Samples from 16 months old mice; Apo0w/Apo16w, Samples from mice on the ApoE-deficient background that were kept on high fat diet for 0 or 16 weeks, respectively; EClu/ECsk/ECbr, EC from lung (lu), skeletal muscle vasculature (sk), or brain (br); LPS, Samples from mice that received 10 mg/kg body weight i.p. 12 hrs before sacrifice.

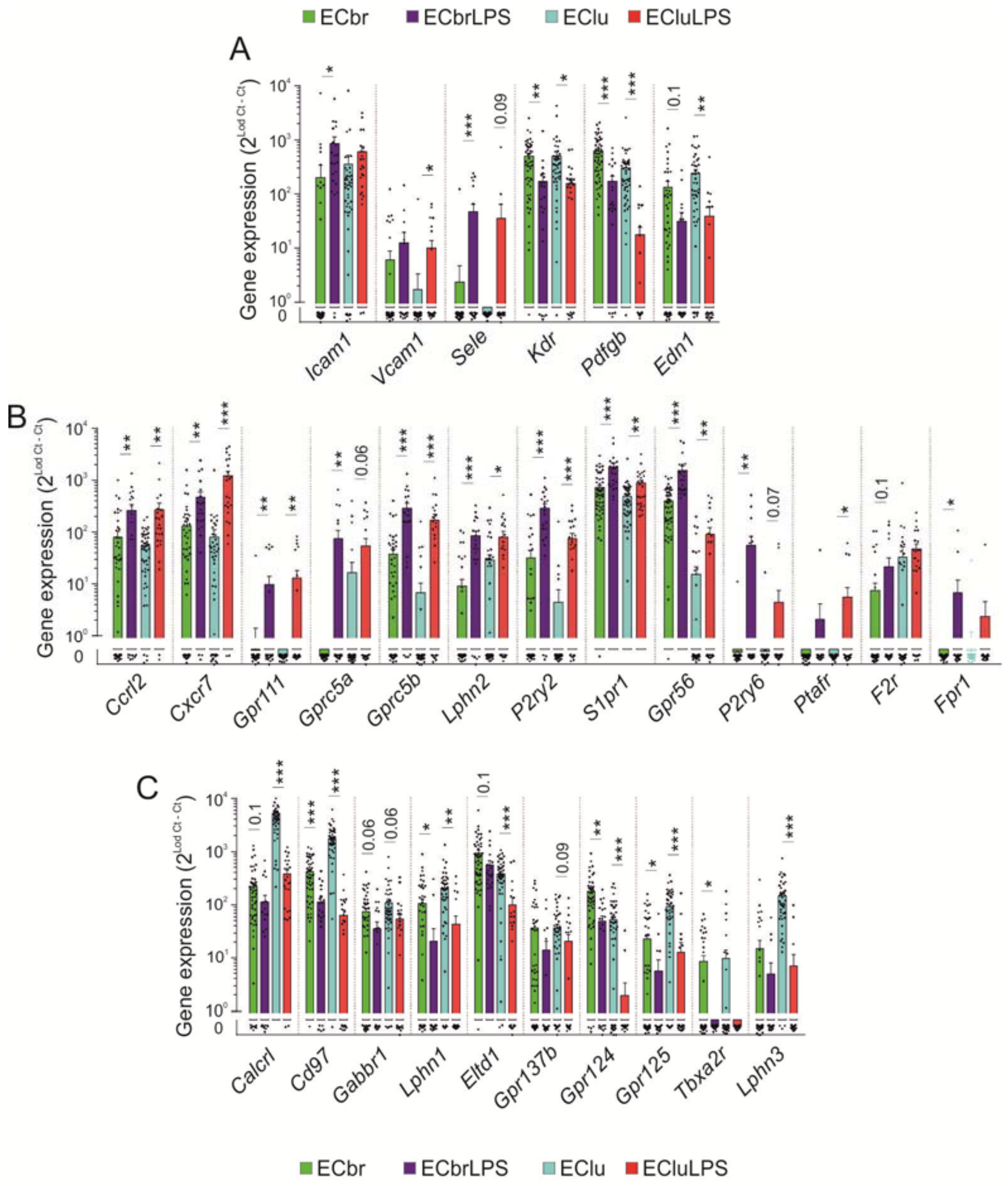

Kaur et al., Suppl. Figure 7

Supplementary Figure 7: Endothelial GPCR pattern after acute inflammatory activation by LPS. A-C, Comparative analysis of expression strength of selected GPCRs in EC from brain (ECbr) or lung (EClu) of healthy or LPS-treated mice: **A,** Function-defining genes upregulated in both EC types upon LPS treatment; **B,** GPCRs upregulated in both EC types upon LPS treatment; **C,** GPCRs downregulated in both EC types upon LPS treatment. All expression data are calculated as $2^{(\text{Limit of detection(Lod) Ct} - \text{sample Ct})}$; LoD C_t was set to 24. Data are means ± SEM; statistical analyses were done by paired t test. *, p<0.05; **, p<0.01; ***, p<0.001.

Supplementary Table 1: Murine primers for single-cell expression analysis		
**Gene name**	**Primary Forward Primer**	**Primary Reverse Primer**
Acta2	GAGGCACCACTGAACCCTAA	TACATGGCGGGGACATTGAA
Actb	CCCTAAGGCCAACCGTGAAA	CAGCCTGGATGGCTACGTAC
Adcyap1r1	GAACGTCAGCAAGAGGGAAA	CTGCAGGATGCATTGGTACAC
Adora1	GTACCTCCGAGTCAAGATCCC	AAACATGGGTGTCAGGCCTA
Adora2a	TACATCGCCATCCGAATTCCA	GAATGACAGCACCCAGCAAA
Adra1a	GAGAAGAAAGCCGCCAAGAC	CTGCAGGATGCATTGGTACAC
Adra1b	ATGTTGGCTCCCCTTCTTCA	CTGCAGGATGCATTGGTACAC
Adra1d	TTCGTCCTGTGCTGGTTCC	CTGCAGGATGCATTGGTACAC
Agtr1a	TAACTCACAGCAACCCTCCA	CTGCAGGATGCATTGGTACAC
Avpr1a	TGTGGTCAGTCTGGGATACC	CTGCAGGATGCATTGGTACAC
Bdkrb2	AAATGCCCTGCTCCTGGAA	CTGCAGGATGCATTGGTACAC
C3ar1	ACTCGTCCAATGCACTTCAC	TGTCAGCATCGAAAGACTCCA
C5ar1	TCGCTGGTTACCACAGAACC	AATGCCATCCGCAGGTATGTTA
Calcrl	ATGCAGGACCCCATTCAACA	CAACGTCATTCCAGCATAGCC
Ccbp2	GAGACCAGATCCTGCAAGCA	AGCTGCTGTTCTCGGAACC
Ccr10	TAGCCAGAGATGGGGACCAA	CGAATAGGCCTCCTCATCGTAC
Ccr1	CCTCAAAGGCCCAGAAACAA	CTCCATCCTTTGCTGAGGAAC
Ccr2	TGCCATCATAAAGGAGCCATA	TAGCACATGTGGTGAATCCA
Ccr3	CTGGACTCATAAAGGACTTAGCA	GTGGTGCCCACTCATATTCA
Ccr4	GACTGTCCTCAGGATCACTTTCA	CCTGGGTGGTGTCTGTGAC
Ccr5	GGAGGTGAGACATCCGTTCC	GGTCGGAACTGACCCTTGAAA
Ccr6	TGGTTCGCCACTCTAATCAGT	GCAGTTCAACCACACTCTCACT
Ccr7	GTGGTGGCTCTCCTTGTCA	GGTATTCTCGCCGATGTAGTCA
Ccr8	AGTGGGCAGCTCTGAAACC	TTGGGCTCCATCGTGTAATCC
Ccr9	CACCATGATGCCCACAGAAC	CTGTGGAAGCAGTGGAGTCATA
Ccrl1	GGGAGATTCAAGATGGCTACCC	GTTCCAGTCTGGCTCCCAAA
Ccrl2	ATCAAGCAACCTGCCTCAAAC	GGGCCACTGTGTAGTTATCCA
Cd4	AAAGGGACACTGCATCAGGAA	CCCATCACCTCACAGGTCAA
Cd19	CCATCGAGAGGCACGTGAA	ACCACTGGGACTATCCATCCA
Cd25/Il2ra	TGCGTTGCTTAGGAAACTCC	CTGGTGTTCAAGTTGAGCTGTA
Cd44	TTCCTTCGATGGACCGGTTA	TACTCGCCCTTCTTGCTGTA
Cd8a	CAGCAAGGAAAACGAAGGCTAC	GCAGCACTGGCTTGGTAGTA
Cd97	TGACCAGCTTTGCCATCCTA	CCCACCTTGGTGATCAATTCC
Cdh1/E-Cad	ATTGCAAGTTCCTGCCATCC	CAGTAGGAGCAGCAGGATCA
Cdh5	AACGAGGACAGCAACTTCAC	TGGCATGCTCCCGATTAAAC
Celsr2	AGCAATGACTGGGACAGCTA	TGGGTTCAGGTCACACACA
Chrm2	CCGGCACTAGGGTGAAGAA	CACAGCTCGGAGTCCTCA
Chrm3	AGTGACCAAGCACGTGACA	AGGAAGAACACACCAACTGGTTA
Cmklr1	GTAACAGACCAGCCAAGGAC	AGTCGTTGTAAGCGTCGTAC
Cnr2	AGGACAAGGCTCCACAAGAC	GATGGGCTTTGGCTTCTTCTAC
Col1a2	GAAAAGGGTCCCTCTGGAGAA	AATACCGGGAGCACCAAGAA
Col3a1	TGCTGGAAAGAATGGGGAGAC	GGTCCAGAATCTCCCTTGTCAC
Crhr2	GTGGACACTTTTGGAGCAGTAC	TCCAAGGTCGTGTTGCAGTA
Csf2/GMCSF	AGTCGTCTCTAACGAGTTCTCC	CCGTAGACCCTGCTCGAATA
Csf2rb	CCTCACCTTGCTCCTGATCC	GGTTGGGGATCTTTTCCTTCCA
Cspg4	GCAGAGGAGGTCTTGGTGAA	GAGGACATCTCGTGCTCATACA
Cx3cr1	TCCTGCAGAAGTTCCCTTCC	GCCTCAGCAGAATCGTCATAC
Cxcr1	CCTCCCGCACACAAGGAA	GCAGCATTCCCGTGATATTTCC
Cxcr2	GCCACTCTGCTCACAAACA	AGGGCATGCCAGAGCTATAA
Cxcr3	ACCAGCCAAGCCATGTACC	GGGAGAGGTGCTGTTTTCCA
Cxcr4	GGTAACCACCACGGCTGTA	CAGGGTTCCTTGTTGGAGTCA
Cxcr5	GGACATGGGCTCCATCACATA	TCCCTCGACTGTAGAGCAGAA
Cxcr6	TGAGCACACTTCACTCTGGAA	ACTCTTGATGCCCATCATCCA
Cxcr7	ACAAACTGCTCAGCACTGAA	GTCTTGAGGAGAGCGACCAA
Cystlr1	GCTTCAGGGAGCAAAAGGAC	GTGGCCACTGTTCTTATGTTGTA
Darc	GCCATGGGGAACTGTCTGTA	AACGAATAATTCCAGCTGTCGAAA
Dll4	GAGAAGGTGCCACTTCGGTTA	TAGAGTCCCTGGGAGAGCAAA
Edn1/ET-1	GACTTTCCAAGGAGCTCCAGAA	GGCTCTGCACTCCATTCTCA
Ednra	CATAGGACCTGCATGCTCAAC	AAAGAGCCACCAGTCCTTCA
Ednrb	CCCTTTCAGAAAACAGCCTTCA	GCGGCAAGCAGAAGTAGAAA
eGFP	AAGGACGACGGCAACTACAA	TTCAGCTCGATGCGGTTCA
Egr1	CCGAGCGAACAACCCTATG	AAGCGGCCAGTATAGGTGAT
Eltd1	AGGACCAGCGTGTCTAATCA	CAGCAGTGTGGCGGAAAA

Emr1	ACAGCTGTACCTGTCAACCA	ACGTCTCGAGTCACACATTCA
Emr4	TCTGCAATAGCTGGCCACAA	GCTCCAGATGAATCCTCGATGAA
F2r	GCGGTCCCTTGCTGTCTT	TCACCGTAGCATCTGTCCTCT
F2rl1	GGCTGCTGGGAGGTATCAC	GTGATTGGAGGCTGGGTTTCTA
F2rl2	CCGGGTCTCAGGACATCAA	GGCTTTGCTGAGTTGTCTGAA
F2rl3	TGATGTAGAGAGTACCAGGGGAA	TCACTGTCGTTGGCACAGAA
Fos	ATGGGCTCTCCTGTCAACAC	GCTGTCACCGTGGGGATAAA
Fpr1	TCTGTCCAGAGCTGTTGGAA	GGTTCACTGCAGACTTGTTCA
Fpr2	TACACCACAGGAACCGAAGA	TCAGATGGATGGAGTAGTTGGA
Gabbr1	AGATCCAGCTGTGCCTGTTAAA	CGGGCTTCGGTCTCATAGAAA
GAPDH	AGACGGCCGCATCTTCTT	TTCACACCGACCTTCACCAT
Glp1r	AGGAACCCTACGCTTCATCAA	CAGTACAGGATAGCCACCATCA
Glp2r	GCCAACAGTGCTTCCTGAAA	GGAATAACAAACAGCATGGGGAA
Gpr107	GTCTCTCTCGTTGGTGTTCCA	TACAACAGCCCAGCCTTCAA
Gpr108	TCCACAACTGTCACAACTCC	TGGATTCTTCTCCCGGATCA
Gpr111	GCAGCTCTTACACAACATCTCAA	TGGTTAGCCATGGCACTGTA
Gpr114	GGAAAATGGCACAGGCTTCC	CCATGACCAGGATGCTGTGTA
Gpr116	AGCCAGAGGCAGTAAGAACA	AGAAATTGGGTCCCGGGTTA
Gpr124	TCACGCTCACCAACTACCAA	ATCCAGAGCAGTGACGACAA
Gpr125	TGGGAGGAATAACCCAGACC	ACCGTGGCAAGGGTAGAATA
Gpr126	TGTGAAGTCGACGTGAACCA	AAGGTGGCTGGCCAACTAAA
Gpr132	TGCCCTGTGCTGCCTCA	CCCTTGTGAAGGTGGCTCTG
Gpr133	TCCGCTTGCCCAATAAATCC	ACCTCACCCACCGTTCTTAA
Gpr137	GCTCCTGGGAGCATAGCC	TCCGATGGCACCATACCAAC
Gpr137b	ATGTCCCTGGCCAACATCTA	CAAGATGACGGTGACACCAA
Gpr153	GCCAACGACGAAGATTCTGAC	AAGAGAGCGCTCCAATACCA
Gpr176	CGTGCAGGTCGTCATCTTCA	GTGCGGCAAGTTGACCATAAC
Gpr18	TCAGACAGCCTTTGACAGACA	CTCAGGGTGGCCATCTTACA
Gpr183	AAACACGGACTGCCACAAC	TTGCCAGTGGGGTAGTGAAA
Gpr19	GGAGCCTGACTCTGAAATCCA	CAGCAGGGTGGCAGTCA
Gpr21	GCATTGCAAGGCTTTCGGTTA	AGCAGGAGTTGTAATGAGTCTCC
Gpr30	CAGCAGATGGCCTGGTAAC	TCCAGATCGCTGCTCTGTTA
Gpr34	AGCTGACACAACCTGGTCTA	ACTTCAGAAGCTGCAGGAAC
Gpr39	CGGACAGCAAGAAGACAGAC	ATCTGATTGGGCATCCAACAC
Gpr4	GTCGGGACCAAGTCAGAGAC	CAGAGTTGATCCTGGTGGAGAC
Gpr56	TCCAGCTTGTCATCCTCTACC	CATGGACCAGTACCACAGGAA
Gpr63	TGTTGTGGGCTATAGCAGGAA	ACACAGCTCCATCTTCCACAA
Gpr64	GTGACAGTCGCACTGAAACA	TTCTGCCCAAGTCCCAGAATA
Gpr65	CAGATTTGCCAGCCTCCTCA	CGCCGTTTGGCATTCTTTCA
Gpr83	TGGCGGACATCATGATCACA	CAAACACCCATGTGCTGTTCA
Gpr97	TCTAGACATTGGTGCAGGGAA	CCACCATGCGGTTGTTCAA
Gprc5a	GGGACACACTCTATGCACCTTA	GCCCTCGGGATAGAGAAATCC
Gprc5b	CGGGCCTACATGGAGAACAA	AGATCTTTGCTCCAAGCTTCCA
Gprc5c	TGCTGACCAGCGTGTACC	ATGACGTCGTATGCACCTTCA
H2-Ab1	CGGTGTGCAGACACAACTAC	GTCCTGGACAGGGAGATGAC
Hey2	GTGGGGAGCGAGAACAATTAC	TGTCGGTGAATTGGACCTCA
Hif1a	TCGACACAGCCTCGATATGAA	TTCCGGCTCATAACCCATCA
Hprt	CAGTACAGCCCCAAAATGGTTA	AGTCTGGCCTGTATCCAACA
Hrh2	GTCCTAAGCGACCCGGTAC	TGGCTACTCTTTCTGGGGTCATA
Htr2a	GCTGCTGGGTTTCCTTGTCA	ACAGAGCTTGCTGGGCAAA
Icam1	AGGGCTGGCATTGTTCTCTA	TGTCGAGCTTTGGGATGGTA
Ifng	GGCACAGTCATTGAAAGCCTA	GCCAGTTCCTCCAGATATCCA
Il10	AAAGGACCAGCTGGACAACA	TAAGGCTTGGCAACCCAAGTA
Il17a	CAGACTACCTCAACCGTTCCA	CACTGAGCTTCCCAGATCACA
Il1b	CTCTCCACCTCAATGGACAGAA	TTGTCGTTGCTTGGTTCTCC
Il2	CGGCATGTTCTGGATTTGAC	CAGTTGCTGACTCATCATCGAA
Il6	CGATGATGCACTTGCAGAAA	ACTCCAGAAGACCAGAGGAA
Itgam	AAGCAGCTGAATGGGAGGAC	GCCCCATTGGTTTTGTGAAACA
Kdr	ATTTCACCTGGCACTCTCCA	TCCCAGGAAAGGGTTTCACA
Klf2	CTAAAGGCGCATCTGCGTAC	TTCCAGCCGCATCCTTCC
Lgr4	CTCAGGCTATTAAAGCCCTTCC	ATGCTCCATCCGGGATAACA
Lgr5	CTCCAACCTCAGCGTCTTCA	ATGTAGGAGACTGGCGGGTA
Lgr6	TATCCGCCACATCCCTGAC	CATGCTGGATGCGGTTGTTA
Lpar1	AGTTCTGGACCCAGGAGGAA	CCGGAGTCCAGCAGACAATAA
Lpar2	GGGTCTCAGTCCTGCTTCAA	AAGCCGATGGTCTCGTTGTA

Lpar3	GAGGGCTCCCATGAAGCTAA	GCAGCAGAACCACCAGAC
Lpar4	AGTGCGAGTTGCCAGTTTAC	CAGACGATCAGAGAGTTCCTCA
Lphn1	TCTCCGCCAACACCATCAA	AAGAGGCCCAGGTTGTTGTA
Lphn2	GAAGACATGCAGGGCTTACC	TCCAGGATTCCAAAGCCTCA
Lphn3	TCGCCTTGACACCTACTTCA	GCAATCCCAAGGAAGATGACA
Ly6g	ACCTGAGACTTCCTGCAACA	AGTAGTGGGGCAGATGGGAA
Lyve1	CAAAGCCTATTGCCACAACTCA	GAGTGTCCAACACGGGGTAA
Mki67	GAGACATACCTGAGCCCATCA	GCTTTGCTGCATTCCGAGTA
Mrgprf	AGATGGCCGGAAACTGTTCA	TCCCGAGCCTCGCTCATA
Myh11	CCCAAGCAGCTAAAGGACAA	AGGCACTTGCATTGTAGTCC
Npy1r	TTCGGCCCACTCTGCTTTATA	GTCCCGGATCTTGTCCATCA
Olfr78	CCTGCTGCTCTCCTTGAGTA	GGAACTCATCGTTGACACCAA
P2ry1	GTTTGTGAAGGCACGAGATCC	CACACACTGGTCTTTTGGTCAA
P2ry10	GCTTGCTAAAGGGGCTTTCTA	CTGTTGCTGCCCATCTTGAA
P2ry12	CTTGATGTGGGCGTACCCTA	GTTCCCAAAGCCCTCTGTGATA
P2ry13	ACAGCTGAGTCTCTTCCAAAAC	CACCGCTCAGACTTGTTGAA
P2ry14	ACACTGATGCCTGGGCTAC	GGCAAGCTTCGTCAACAGAA
P2ry2	GAGCATCCTCACCACCTCAA	GGTGCTATTCCAGGGTTCCA
P2ry6	CCAAATCTGGCACTTCCTCCTA	CTGCCTGTCAGCCTTTCCTA
Pdgfb	CGGCCTGTGACTAGAAGTCC	TCACCGTCCGAATGGTCAC
Pecam1	AGAGCCAGCAGTATGAGGAC	GCAATGAGCCCTTTCTTCCA
Prokr1	TCAGAAGACAGAGGTGCTCCTA	GGACCCGGTATCTGAGTTCC
Ptafr	AATTGCCAGGCCACAACAC	TGGTCATGGAGCGCTGAATA
Ptger1	CATGGGGACCCTGCATCC	CATCCGCTAGGCTCAGGTTAA
Ptger2	TGACCATCACCTTCGCCATA	TCGGAGGTCCCACTTTTCC
Ptger3	CATCCAGCTCATGGGGATCA	GCTCAACCGACATCTGATTGAA
Ptger4	GTGCTCATCTGCTCCATTCC	CAATCCTGATGGCCTGCAAA
Ptgfr	CTCCGGACACAACCACTCA	AAAAGCTGTCCCCTCAAGTCA
Ptgir	ATTCTGCTGGCCCTCATGAC	GGCGATGGCCTGAGTGAA
Pth1r	TGGGCACAAGAAGTGGATCA	AGCACCCGGATGATGTTGATA
Ptprc/CD45	TGATGAGGGCAGACTGTTCC	TCGGGCATCTTTGATGGGAA
Rorc/RORgt	TGGAGCTCTGCCAGAATGAC	GGCCCTGCACATTCTGACTA
S1pr1	CGGTGTAGACCCAGAGTCC	GAGAGGCCTCCGAGAAACA
S1pr3	GCCCCTAGACGGGAGTCTTA	ACTGCGGGAAGAGTGTTGAAA
Sele	AAAGACTCGGGCATGTGGAA	CTGCAGGATGCATTGGTACAC
Smtn	ACCGACGCCAGAACTTTGAA	CGCACCATGTCCTCTGTATCC
Sucnr1	AGCTCCTGGCAGAGTTTTCT	TCGATTGCATAAAATGCAGAGAGG
Tbx21/Tbet	CAAGTTCAACCAGCACCAGAC	CCACGGTGAAGGACAGGAA
Tbxa2r	TTCAGCTCGTGGGCATCA	CAGGTGGTGTCTGCAACAAA
Tgfb1	GCTGCGCTTGCAGAGATTAA	GTAACGCCAGGAATTGTTGCTA
Tnf	GGGTGATCGGTCCCCAAA	TGAGGGTCTGGGCCATAGAA
Tnni2	ATGGAGGTGAAGGTGCAGAA	ACTTGCCCCTCAGGTCAAATA
Vcam1	CCCAAACAGAGGCAGAGTGTA	CTGCAGGATGCATTGGTACAC
Vipr1	TGGACGATTGTCAGGATCCA	ATGGGGCCCTTTATGATCCA
Vipr2	GCCTGGTGGTAGCAGTTCTATA	GCCTCGCCATCTTCTTTTCA
Xcr1	GAAACCCTGACATGGACTCA	AAAGGCTGTAGAGGACTCCA

Supplementary Table 1: Primer sequences for the detection of murine genes by single-cell RT-PCR.

Supplementary Table 2: Human primers for single-cell expression analysis		
**Gene name**	**Primary Forward Primer**	**Primary Reverse Primer**
ACTA2	ACCCGATAGAACATGGCATCA	GGCAACACGAAGCTCATTGTA
ADRA1B	TCATCTTGTGCTGGCTACCC	GGCTTCAGGGTGGAGAACA
ADRA1D	GAGAAGAAAGCGGCCAAGAC	GCCCTCCGATGGCTTCA
CALCRL	ACAACCAGGCCTTAGTAGCC	ACAGCCCATCAGGTAAAGATGAA
CD97	GGGCAGAGACTCCAAGACAA	AGGAGCTTCCATCAGTTCATCC
CELSR2	TGGCTGACTTCATTGCCAAC	GTGGCAAGTGTTGCTGTCA
EDNRA	TGAGAATTGCCCTCAGTGAACA	AGGGAACCAGCAAAGAGCAA
EDNRB	TGCACATCGTCATTGACATCC	GAAAGGCACCAGCTTACACA
F2R	CGCCTGCTTCAGTCTGTG	GGTGGCATTTGTTGCTTTTGA
F2RL1	TGCTAGCAGCCTCTCTCTCC	GGATGTGCCATCAACCTTACCA
GABBR1	CAATACCCGCAGCATTTCCAA	GTCTCCTCAGGGTGTCTTTTCA
GAPDH	GAACGGGAAGCTTGTCATCAA	ATCGCCCCACTTGATTTTGG
GPR107	CAAGTCTCTTTCCTTGGTGTTCC	AACAGCCCAGCCTTCGATA
GPR108	CAGTTTCCTGGTCCTGTTCC	AACAACGTCTTCTGCTCTCC
GPR124	TCACCTCCGAGACCTTCCA	CCAGGTTGCAGACTGGAGAA
GPR125	TGTGGGAGGAATAACCCAGAC	ACTGTGGCAAGGGTGGAATA
GPR126	GAAAGGTTTCAATGCCAGCTAC	AGCATCTGATGTCTGGGGTA
GPR137	GTGGTGTTCAAGGCCAAGGTG	ACAAAGGCCCCTCGGACAG
GPR137B	TTTCAAAGCCAAGTCAAAATATTCTCCA	ATGAAGAGGGAGGCCAGGTA
GPR153	GGCCTCGTGACCACCATA	CAGGCTGCTGAAGCTCAC
GPR30	AGACTGTGAAATCCGCAACC	CTGGAGGTGCACTTGGAAC
GPR56	CCAGCTTGTCGTCCTCTACC	GCATGGACCAGTACCAGATGAA
GPR64	TCCTCCCCAGAAGAGTTGGAA	TGGATGGTCAGCAAGACAGAC
GPRC5B	AGCAACGTGTATCAGCCAAC	CAAAGGTGTCTTCCTGTGTGAC
GPRC5C	TCAGAGCATGTTCGTGGAGAA	GTACCCGCTGTATGGTGACA
ICAM1	CCCCTACCAGCTCCAGACC	TGCGTGTCCACCTCTAGGAC
IL6	AGAGCTGTGCAGATGAGTACAA	GTTGGGTCAGGGGTGGTTA
LGR4	TTGCCACACTTGGGCCAATA	CATTCAGGCCTTCCGTAGGAAA
LPAR1	GGCGGAATCGGGATACCA	ACAGCACACGTCTAGAAGTAACA
LPHN1	GCTGTCTGCCAAAACCATCA	AAGAGGCCCAGGTTGTTGTA
LPHN2	CTGTCCGCAAATACCGTCAAA	CAGGCTCCGGTAAATGATGAAC
LPHN3	ACTCGTCAAGGACAGATAGCA	TCCATCAGGAGCAAGGCATA
MRGPRF	CCATCCCGGCAACAGGAA	GATCTGCTCGATGGTCAGGAA
P2RY2	TCCAGGCGTGTGCATTCA	AGGTGCCATTGATGGTGTCA
P2RY6	GCTTTGGAAGGCGGAGTTCA	TGCCCAGGTGGGTTTCCTA
PTGER4	GTGCTCATCTGCTCCATCCC	CTCGCTCCAAACTTGGCTGATA
PTGIR	AAGCAAGTGAAGGCACAGAC	GCACGAATCCGCCATCC
PTH1R	TCAGGCCCAGTGCGAAAA	GTCCATCCCTTGTCTGATTCCA
S1PR1	AAGCGAGCCGTACAGATCC	GAGAGGGCCTCGGAAACC
TBXA2R	CTCAGCTCCTGGGGATCA	AGATGAGCAGCTCCTTCTCC

Supplementary Table 2: Primer sequences for the detection of human genes by single-cell RT-PCR.

Supplementary Table 3: Predicted and observed melting temperatures in sc RT-PCR				
Gene name	predicted TM	Observed TM_EC	Observed TM_SMC	Observed TM_Leukocytes
Acta2	82.46	82	82	no response
Actb	83.05	83	83	83
Adcyap1r1	83.62	83	83	83
Adora1	88,18	no response	87	87
Adora2a	84.83	84	84	84
Adra1a	86.95	86	86	86
Adra1b	86.97	86	86	no response
Adra1d	84.33	84	84	84
Agtr1a	80,6	81	81	81
Avpr1a	83.61	no response	83	no response
Bdkrb2	87,1	86	86	86
C3ar1	85,0	no response	no response	85
C5ar1	83,5	82	82	82
Calcrl	82.87	82	82	82
Ccbp2	87,4	86	86	no response
Ccr1	80.04	80	no response	80
Ccr10	84,9	no response	no response	84
Ccr2	82,7	no response	82	82
Ccr3	80.67	no response	no response	80
Ccr4	86,0	no response	no response	85
Ccr5	82,5	82	82	82
Ccr6	80,36	no response	81	81
Ccr7	82,0	82	no response	82
Ccr8	82,9	no response	no response	82
Ccr9	80,9	no response	no response	81
Ccrl1	83.34	no response	84	84
Ccrl2	84,0	83	83	83
Cd19	84,6	no response	no response	83
CD25	83.58	83	83	83
Cd4	81,9	no response	no response	81
CD44	80.08	80	80	80
Cd8a	82,4	no response	no response	83
Cd97	83,1	82	82	82
Cdh5	83.22	83	no response	no response
Celsr2	79.39	79	79	79
Chrm2	87,78	87	87	no response
Chrm3	79.55	79	79	79
Cmklr1	82,1	82	82	82
Cnr2	86,8	no response	86	86
Col1a2	86.75	86	86	86
Col3a1	86,5	85	85	85
Crhr2	82.72	81	81	no response
Csf2	81.02	81	81	81
CSf2rb	82,0	81	81	81
Cx3cr1	83.24	no response	82	82
Cxcr1	84.25	no response	no response	84
Cxcr2	80.73	81	no response	81
Cxcr3	83,1	no response	82	82
Cxcr4	82,3	82	82	82
Cxcr5	83,0	82	no response	82
Cxcr6	80,8	no response	81	81
Cxcr7	87,5	87	87	87
Cysltr1	80,65	80	80	80
Darc	79.48	79	79	no response
Dll4	81.34	81	81	no response
Edn1	84.2	84	84	84
Ednra	81.14	81	81	81
Ednrb	80.52	80	80	80
eGFP	86.88	87	87	87
Egr1	85.92	85	85	85
Eltd1	78.31	78	78	78
Emr1	80.05	80	80	80
Emr4	81,78	81	81	81
F2r	84,4	83	83	83
F2rl1	87.51	87	87	87
F2rl2	83,3	83	no response	83

**Supplementary Table 3 (continued): Predicted and observed melting temperatures in sc RT-PCR**				
Gene name	predicted TM	Observed TM_EC	Observed TM_SMC	Observed TM_Leukocytes
F2rl3	86,8	86	86	86
Fos	83.36	83	83	83
Fpr1	80,5	80	no response	80
Fpr2	80.78	81	no response	81
Gabbr1	81,5	81	81	81
Gapdh	83.89	84	84	84
Glp1r	81.69	81	81	81
Glp2r	86,07	no response	86	no response
Gpr107	82.7	82	82	82
Gpr108	83.88	83	83	83
Gpr111	79.33	79	79	no response
Gpr114	82.84	82	82	82
Gpr116	80.98	80	80	80
Gpr124	82.88	82	82	82
Gpr125	81.9	81	81	81
Gpr126	84.64	84	84	84
Gpr132	84.24	83	83	83
Gpr133	83.36	83	83	83
Gpr137	88.28	87	87	87
Gpr137b	82.12	82	82	82
Gpr153	82.83	82	82	82
Gpr176	81,3	81	81	81
Gpr18	82,9	no response	no response	82
Gpr183	82,8	83	83	83
Gpr19	84.42	84	84	84
Gpr21	80.96	81	81	no response
Gpr30	82.93	83	83	no response
Gpr34	80.43	80	80	80
Gpr39	81,74	no response	82	no response
Gpr4	88.31	88	88	no response
Gpr56	80.56	80	80	80
Gpr63	79.3	80	80	no response
Gpr64	80,23	81	81	no response
Gpr65	81.61	81	no response	81
Gpr83	82,5	82	82	82
Gpr97	84.95	84	no response	84
Gprc5a	79.42	78	78	78
Gprc5b	83.64	83	83	83
Gprc5c	86.28	86	86	86
H2-Ab1	88,5	87	87	87
Hey2	82.23	82	82	82
Hif1a	76,4	77	77	77
Hprt	81,7	81	81	81
Hrh2	86,6	86	86	86
Htr2a	85.36	84	84	84
Icam1	84,7	84	84	84
Ifng	78,3	no response	no response	78
Il10	80,7	81	81	81
Il17a	83.09	83	no response	83
Il1b	78.34	78	78	78
Il2	80.54	no response	no response	81
Il6	78.65	78	78	78
Itgam	81,6	no response	no response	80
Kdr	79.59	79	79	79
Klf2	82.07	82	82	82
Lgr4	79	78	78	78
Lgr5	82.17	no response	82	no response
Lgr6	82.8	82	82	no response
Lpar1	84,3	84	84	84
Lpar2	89,1	no response	88	88
Lpar3	86.43	86	86	no response
Lpar4	80.75	81	81	no response
Lphn1	83,3	83	83	83
Lphn2	81.65	82	82	82
Lphn3	79,24	79	79	no response
Ly6g	84.15	no response	no response	85

Supplementary Table 3 (continued): Predicted and observed melting temperatures in sc RT-PCR				
Gene name	predicted TM	Observed TM_EC	Observed TM_SMC	Observed TM_Leukocytes
Mki67	79,76	80	80	80
Mrgprf	84.09	83	83	no response
Myh11	80.88	no response	80	no response
Npy1r	78.17	78	78	no response
Olfr78	86.81	86	86	no response
P2ry1	81,2	81	81	81
P2ry10	81.03	80	no response	80
P2ry12	82.57	82	82	82
P2ry13	83.35	83	no response	83
P2ry14	84,9	no response	84	84
P2ry2	85,4	84	84	84
P2ry6	80,8	80	80	80
Pdgfb	87.17	87	87	87
Pecam1	85.04	84	84	84
Prokr1	86.19	85	85	85
Ptafr	88,0	87	87	87
Ptger1	87.67	86	86	no response
Ptger2	82,2	81	81	81
Ptger3	81.39	no response	81	81
Ptger4	83,4	83	83	83
Ptgfr	84,77	84	84	no response
Ptgir	86,8	86	86	86
Pth1r	83.34	83	83	no response
Ptprc	82.15	no response	no response	81
Rorc	81.92	81	81	81
S1pr1	87,0	87	87	87
S1pr3	86,5	85	85	85
Sele	81.8	82	82	82
Smtn	86.71	86	86	86
Sucnr1	81.82	no response	82	no response
Tbx21	81.81	81	81	81
Tbxa2r	85.85	85	85	85
Tgfb1	78.95	79	79	79
Tnf	83.86	83	83	83
Vcam1	82.25	82	82	82
Vipr1	83.32	83	83	83
Vipr2	80.25	80	80	80
Xcr1	82.74	82	no response	no response

Supplementary Table 3: Predicted and observed melting temperatures (TM) of single-cell amplificates in individual endothelial cells (EC), smooth muscle cells (SMC), and leukocytes.

Cell type	No. of cells	No. of mice	No. of exps
SMao	60	8	3
SMaoAged	32	6	2
SMaoApo0w	34	6	2
SMaoApo16w	50	6	2
SMsk	57	7	3
SMmes	29	8	5
SMub	25	8	4
ECsk	40	6	5
EClu	48	8	3
ECluLPS	25	4	2
ECbr	52	7	2
ECbrLPS	22	4	2
ECao	12	4	2
ECaoApo16w	16	6	2

Kaur et al., Suppl. Table 4

Supplementary Table 4: Overview of numbers of cells, mice, and independent experiments analyzed within this study.

Supplementary Table 5

TF_NAME	GPCRs upreg. in SMaoApo16wk	GPCRs downreg. in SMaoApo16wk
Arid3a	-1,66042	-1,06226
Arid3b	-2,80635	-1,14491
Arid5a	-2,99473	-1,38864
Atoh1	0,159265	3,27371
Barhl1	-1,2229	-2,31807
BARHL2	-1,43869	-2,16393
BARX1	-1,2474	-1,89493
Bcl6	1,77499	-1,29264
BCL6B	1,6166	-1,60665
BHLHE23	-1,65292	-0,276239
BSX	-1,12441	-2,24383
CDX1	-1,75533	-2,53328
CDX2	-1,58111	-1,63113
Crem	-1,92517	0,363056
CTCF	0,783412	2,5925
CUX1	-1,95353	-1,23749
CUX2	-2,09452	-1,12924
Dlx1	-1,36268	-2,59683
Dlx2	-2,07165	-2,65772
Dlx3	-1,93016	-2,40513
Dlx4	-1,7411	-2,29646
DLX6	-1,80725	-2,32859
Dmbx1	-2,333	-0,432666
DMRT3	0,124848	-1,63975
Dux	-2,82348	1,37919
DUXA	-1,96425	-0,912171
E2F1	2,40256	2,1137
E2F3	2,06549	2,29237
E2F4	2,38808	1,24331
E2F6	1,51972	2,25736
EBF1	0,803425	1,75768
EGR1	2,76929	2,32403
EGR2	2,54954	1,5261
EGR3	2,74859	1,81394
EGR4	2,60641	1,60401
EHF	-0,260697	1,88148
ELK3	-0,826669	-1,63982
EMX1	-0,826746	-1,9911
EMX2	-1,20462	-1,80267
ESR1	1,85348	2,41763
ESR2	2,19876	1,49262
ESRRA	0,103269	2,10687
Esrrb	0,0523782	1,8089
ESX1	-1,83964	-1,58704
ETV4	-1,67787	-2,04186
ETV5	-1,30401	-1,82117
EVX1	-2,02224	-1,54713
EVX2	-1,7379	-1,56461
EWSR1-FLI1	0,507374	3,23305
FOS::JUN	-1,95706	-0,0930281
FOSL1	-1,89914	1,34901
FOXB1	-2,0449	-1,59857
FOXC2	-3,17709	-0,802811
Foxd3	-2,75508	0,345906
FOXF2	-0,0718657	-1,60607
Foxj3	-2,30151	-0,852913
Foxk1	-3,02098	-1,30365
Foxo1	0,814846	1,62577
Foxq1	-2,68402	-0,48225
GATA5	-0,95304	-2,20841

GBX1	-1,35779	-2,06819
GBX2	-1,93985	-2,3279
GLIS3	-1,65795	1,03952
GRHL1	-2,26336	-1,8629
GSC	-3,0533	-1,29858
GSC2	-2,80069	-0,780735
GSX1	-1,16427	-2,33833
GSX2	-1,50367	-2,06674
HESX1	-1,77108	-1,94857
HINFP	2,33755	-0,197054
Hmx1	-2,30191	-2,33781
Hmx2	-2,77302	-2,07255
Hmx3	-2,8367	-1,87095
HNF4A	1,66532	0,956611
HOXA10	-1,8021	-1,1682
HOXA2	-0,797026	-1,64996
HOXA5	-1,65617	0,170172
Hoxa9	-1,66114	-0,394397
HOXB13	-1,83452	-1,16878
HOXB2	-1,67164	-2,58202
HOXB3	-1,68329	-2,40705
HOXC10	-0,642064	-1,84443
HOXC11	-1,21585	-1,89657
HOXC12	-1,17372	-1,70232
Hoxc9	-2,29962	-1,36576
HOXD12	-1,83087	-1,10901
HOXD13	-1,85636	-1,72701
Hoxd8	-1,98401	-1,8564
Hoxd9	-1,66456	-1,96536
HSF1	2,23923	-1,14477
HSF2	2,25417	-1,48421
HSF4	2,0409	-0,944961
INSM1	2,70537	-0,714727
ISL2	-0,146881	-2,33572
ISX	-1,43794	-2,55095
JDP2	-1,8949	0,951988
JUN(var.2)	-1,75143	0,291464
JUND	-2,36211	1,26293
KLF13	0,451152	1,91772
KLF16	1,63285	1,51014
Klf4	2,16242	1,2901
KLF5	2,91954	0,906865
LBX2	-1,61285	-1,85421
LHX2	-1,18482	-1,69377
Lhx3	-1,82908	-1,49732
Lhx4	-1,84326	-1,39275
Lhx8	-2,92589	-0,72724
LHX9	-1,56389	-2,46537
LIN54	-2,39634	-0,0746805
LMX1A	-2,3781	-1,39864
LMX1B	-2,84384	-1,63724
MEOX2	-1,97795	-2,32387
mix-a	-1,67699	-2,41297
MIXL1	-1,97055	-1,36952
MNX1	-1,34094	-1,87043
MSX1	-1,27496	-1,76097
MSX2	-1,37784	-1,98824
Msx3	-1,80058	-2,42557
MYBL2	-1,8613	-0,566999
MZF1	2,18474	0,531378
Neurog1	-2,06399	1,33154
NFATC1	-1,66037	-0,552214
NFATC3	-1,86784	-0,642091
NFE2	-1,35165	1,92951

Nfe2l2	-1,61852	1,37563
NFIL3	-1,84663	-1,88713
NFKB1	2,61273	1,42237
NFKB2	2,43297	0,106463
NHLH1	1,88906	0,914811
Nkx2-5	-1,94113	-1,47575
NKX6-2	-0,731172	-2,01568
Nobox	-1,46488	-2,26476
NOTO	-1,24178	-1,80342
NR2C2	1,61093	1,69917
Nr2f6(var.2)	-0,38395	2,02195
NR4A2	1,40673	2,24653
OLIG2	-2,15937	-0,118255
OLIG3	-2,14232	0,329689
ONECUT1	-2,53284	-1,20499
ONECUT2	-2,48915	-1,0938
ONECUT3	-2,67718	-1,14386
OTX1	-2,29231	-1,95674
OTX2	-2,38857	-1,6459
PAX1	0,426337	1,67527
PAX3	-4,15709	-1,37474
Pax5	1,38689	2,50655
PAX7	-4,15448	-1,29422
PAX9	1,23573	1,76295
PBX1	-2,55444	0,618404
PDX1	-1,67677	-2,47493
PHOX2A	-2,47834	-0,663167
Phox2b	-2,262	-0,583335
Pitx1	-2,27666	-1,33982
PITX3	-2,19962	-1,4975
PLAG1	2,68678	1,65661
POU4F1	-2,78631	-0,54976
POU4F3	-2,33336	-0,733161
POU6F1	-1,60619	-2,86244
POU6F2	-1,62545	-0,976179
PPARG	1,77877	1,99952
PROP1	-2,49155	-1,57833
PRRX1	-2,27025	-1,79306
Prrx2	-2,04564	-1,86516
RARA(var.2)	2,75778	0,124374
Rarb(var.2)	2,54286	0,830734
RAX	-2,58011	-1,84518
RAX2	-1,7109	-2,00099
REST	2,35128	1,51328
RHOXF1	-1,79606	-0,445131
RORA	0,378917	2,79388
SCRT2	0,776652	1,89768
SHOX	-2,09574	-1,92242
Shox2	-1,63379	-2,28384
SMAD3	1,8889	0,744229
Sox1	-0,707282	-2,0378
SOX10	-1,24752	-2,82919
Sox17	-0,762902	-2,42986
Sox6	-2,604	-1,1446
SOX9	-2,46029	0,0757434
SP1	2,193	1,47957
SP2	2,33445	1,25918
SP3	1,91901	1,24998
SPI1	1,37039	1,82539
Spz1	2,49849	1,93361
SREBF1	0,131635	2,02613
SRY	-2,00102	0,0618808
Stat5a::Stat5b	0,799009	-2,43325
T	-0,729107	1,86384

Tal1::Gata1	0,263152	-2,15699
TAL1::TCF3	-1,09652	2,21074
TBP	-0,56063	-2,23716
TBX1	-2,04405	1,10629
TBX15	-2,125	0,61247
TBX19	-1,17402	1,80195
TBX20	-1,6081	-0,352995
Tcfcp2l1	3,04705	1,02497
TFAP2A	3,17636	1,71592
TFAP2A(var.2)	1,763	1,71977
TFAP2A(var.3)	2,97783	1,03265
TFAP2B	1,91231	1,79516
TFAP2B(var.2)	2,00702	0,0254864
TFAP2B(var.3)	2,70971	1,27491
TFAP2C	3,10663	1,81618
TFAP2C(var.2)	1,81421	0,0620633
TFAP2C(var.3)	2,89025	1,24468
THAP1	2,4821	2,16535
TP53	1,74603	1,45695
TP63	1,71789	-0,0938665
Twist2	-1,72838	-1,35125
UNCX	-2,06674	-1,73997
VAX1	-1,72645	-1,6882
VAX2	-1,66758	-1,54332
VENTX	-2,22074	-1,90058
VSX1	-1,51097	-1,71258
VSX2	-1,28303	-1,6029
YY1	-1,60827	0,502389
ZBED1	1,53325	-1,73396
ZBTB18	-1,30324	2,21998
ZEB1	2,33696	0,497446
ZNF263	2,03006	3,35437

Supplementary Table 5: Full list of TF binding sites significantly ($p<0.05$) enriched (red) or depleted (green) in the promoters (-950 to 0 bp relative to translational start) of GPCRs downregulated or upregulated in SMaoApo16wk compared to a global promotor background set. Color scale represents a rank-based z score.
